# NKX6.3 controls gastric differentiation and tumorigenesis

**DOI:** 10.18632/oncotarget.4952

**Published:** 2015-07-22

**Authors:** Jung Hwan Yoon, Won Suk Choi, Olga Kim, Sung Sook Choi, Eun Kyung Lee, Suk Woo Nam, Jung Young Lee, Won Sang Park

**Affiliations:** ^1^ Department of Pathology, College of Medicine, The Catholic University of Korea, Seocho-gu, Seoul, Korea; ^2^ College of Pharmacy, Sahmyook University, Hwarangro, Nowon-gu, Seoul, South Korea; ^3^ Department of Biochemistry, College of Medicine, The Catholic University of Korea, Seocho-gu, Seoul, Korea; ^4^ Department of Functional RNomics Reasearch Center, College of Medicine, The Catholic University of Korea, Seocho-gu, Seoul, Korea

**Keywords:** NKX6.3, differentiation, cell proliferation, cell death, stomach

## Abstract

NKX6.3 transcription factor is known to be an important regulator in gastric mucosal epithelial differentiation. The present study aimed to investigate whether NKX6.3 acts as an essential tumor suppressor in gastric carcinogenesis. Absent or reduced protein expression and decreased DNA copy number and mRNA transcript of the NKX6.3 gene were frequently observed in gastric cancers. Overexpression of NKX6.3 in AGS^NKX6.3^ and MKN1^NKX6.3^ cells markedly arrested cell proliferation by inhibiting cell cycle progression and induced apoptosis through both death receptor- and mitochondrial-pathways. In addition, stable NKX6.3 transfectants increased the expression of gastric differentiation markers, including SOX2 and Muc5ac, and decreased the expression of intestinal differentiation markers, CDX2 and Muc2. In ChIP-cloning and sequencing analyses, NKX6.3 coordinated a repertoire of target genes, some of which are clearly associated with cell cycle, differentiation and death. In particular, NKX6.3 transcriptional factor was found to bind specifically to the upstream sequences of GKN1, a gastric-specific tumor suppressor, and dramatically increase expression of the latter. Furthermore, there was a positive correlation between NKX6.3 and GKN1 expression in non-cancerous gastric mucosae. Thus, these data suggest that NKX6.3 may control the fate of gastric mucosal cells and function as a gastric tumor suppressor.

## INTRODUCTION

Generally, the gastrointestinal epithelium is characterized by a very high cellular turnover rate, which leads to epithelial renewal every 3-5 days, and apoptosis is a key regulator of this turnover [1]. Pluripotent stem cells occupy a niche in the isthmus or neck region of the gastric glands. Post-mitotic cells migrate up or down from the neck region toward the gland and differentiate into a variety of cell types [2]. Recent data showed that gastric mucosal inflammation is generally believed to be caused by chronic *Helicobacter pylori* (*H. pylori*) infection and atrophic gastritis, while intestinal metaplasia and dysplasia represent different stages of the gastric carcinogenesis cascade [3]. Even though numerous advances in the understanding of gastric cancer have been made, the gastric cancer still remains one of the malignancies with the highest incidence and mortality rates worldwide [4, 5].

Many homeodomain transcription factors play pivotal roles in cell development and differentiation. Among the homeobox genes, NKX family members are involved in a variety of developmental processes, such as cell fate determination in the central nervous system, gastrointestinal tract, and pancreas [6]. NKX6.3, a third member of the NKX6 subfamily of *NKX* gene, is expressed in the epithelium of the most distal stomach and eventually segregates to the lower/base region of the gastric unit [6, 7]. NKX6.3 is predicted to encode a 266-amino acid protein and is located in chromosome 8p11.21 [6]. Since NKX6.3 is expressed in post-mitotic differentiated migrant cells of gastric units and loss of heterozygosity at chromosome 8p11 has been frequently detected [7, 8], we hypothesized that alteration of the *NKX6.3* gene may lead to abnormal differentiation and homeostatic imbalance of gastric mucosal epithelium and may eventually cause gastric cancer.

In this study, we demonstrated that NKX6.3 may play a key role in gastric carcinogenesis by affecting the processes of differentiation, proliferation, and apoptosis of gastric mucosal epithelium.

## RESULTS

### Reduced NKX6.3 protein expression in gastric cancer cell lines and tissues

**Figure 1:**
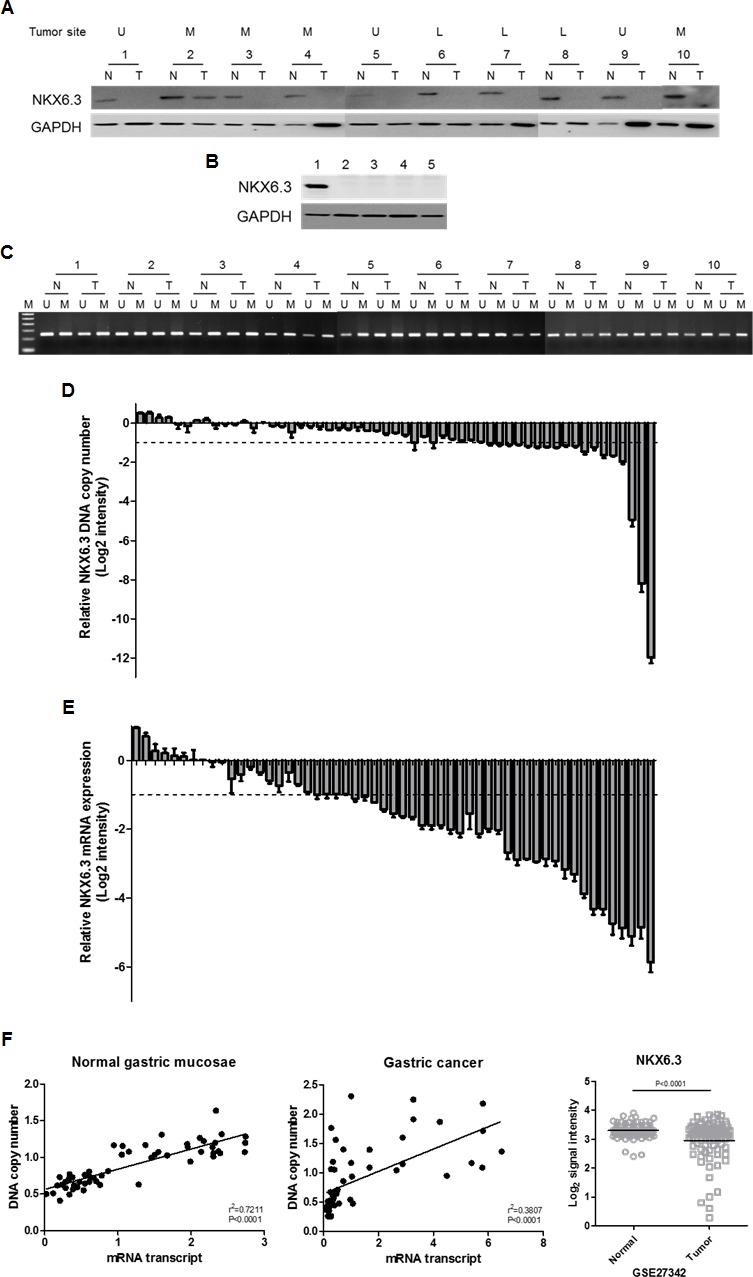
The NKX6.3 expression in gastric cancer cell lines and tissues **A.** Most gastric cancer tissues demonstrated loss or reduced expression of the NKX6.3 protein. U, upper third of stomach; M, middle third; L, lower third. N, corresponding non-cancerous gastric mucosa; T, gastric cancer. **B.** AGS, MKN1, MKN28, and MKN45 gastric cancer cell lines showed no NKX6.3 expression, whereas marked expression of the NKX6.3 protein was detected in AGS cells transiently transfected with *NKX6.3*. Lane 1; NKX6.3-transfected AGS cells, Lane 2-5; AGS, MKN1, MKN28 and MKN45 cells. **C.** Methylated and unmethylated DNAs for the *NKX6.3* gene were found in all gastric cancer tissues and in corresponding non-cancerous gastric mucosa. **D.** and **E.** Fold changes of *NKX6.3* DNA copy number (D) and mRNA expression (E) in gastric cancer compared to corresponding non-cancerous gastric mucosa were assessed by real time QPCR. The result for each patient is represented by scale bar (log2 intensity). **F.** There was a positive correlation between DNA copy number and mRNA transcript expression of *NKX6.3* in corresponding non-cancerous gastric mucosa (left panel) and gastric cancers (middle panel) (linear regression correlation test, *P* < 0.0001). Recapitulated *NKX6.3* gene expression level in the large cohort of gastric cancer patients (NCBI GEO database, accession numbers GSE27342). The relative expression level of *NKX6.3* mRNA in non-cancerous (Normal) and gastric cancer (Tumor) tissues is illustrated by scatterplot. The median expression level of each group is indicated by horizontal lines. Gene expression levels are shown on the ordinate (log2 intensity). The differential *NKX6.3* expression for these two categories was determined by the unpaired *t*-test (*p* < 0.0001; two-tailed) (right panel).

The NKX6.3 protein was found in 35 non-cancerous gastric mucosal tissues including fundus, corpus, and antrum, and its expression was lost or reduced in 33 (94.3%) of 35 gastric cancers (Figure 1A). Additionally, AGS, MKN1, MKN28 and MKN45 gastric cancer cells also showed no expression of the NKX6.3 protein, whereas its marked expression was detected in AGS cells transiently transfected with *NKX6.3* (Figure 1B), confirming tissue data and suggesting a role for NKX6.3 as a tumor suppressor.

### Mutations and methylation status of the *NKX6.3* gene in gastric cancers

The presence of mutation, possibly associated with reduced or loss of NKX6.3 expression, was examined by sequencing analysis. Unexpectedly, none of the *NKX6.3* mutations were detected in 55 gastric carcinomas (data not shown).

We next assessed the methylation status of the *NKX6.3* gene in 55 paired non-cancerous gastric mucosa and gastric cancer tissues. Unexpectedly, all cancer and corresponding gastric mucosa cases showed both methylated and unmethylated DNAs for the *NKX6.3* gene (Figure 1C).

### DNA copy number and mRNA expression of the *NKX6.3* gene were reduced in gastric cancers

In real time-QPCR analysis, the copy number of the *NKX6.3* gene was reduced in 18 (32.7%) of 55 gastric cancer DNAs, compared to the surrounding gastric mucosa DNAs (Figure 1D). We also examined allelic loss of the *NKX6.3* gene in 35 gastric cancers with microsatellite markers D8S464 and D8S2329, which are located −77.692 kb and +3.659 kb from the *NKX6.3* locus, respectively. We found that 18 (51.4 %) of 35 cases were informative at D8S2329 marker and 10 (55.6%) of them showed loss of heterozygosity (Supplementary Figure 1). For D8S464, 12 (34.3%) cases showed heterozygosity and 4 (33.3%) of them revealed loss of heterozygosity (Supplementary Figure 1). In addition, 23 (65.7%) cases were informative at D8S464 and/or D8S2329 markers, and 12 (52.2%) of them showed allelic loss at one or both markers, suggesting that reduced DNA copy number at the *NKX6.3* locus is frequent in gastric cancers.

All corresponding non-cancerous gastric mucosae expressed the *NKX6.3* gene transcript, and the loss or reduced expression of mRNA transcript was observed in 34 (61.8%) of the 55 gastric cancer tissues analyzed (Figure 1E). There was a positive correlation between DNA copy number and mRNA transcript of the *NKX6.3* gene in both non-cancerous gastric mucosae and cancer tissues (*P* < 0.0001) (Figure 1F). To further confirm our results, we recapitulated the *NKX6.3* gene expression from the large cohorts of gastric cancer patients that are available from the National Center for Biotechnology Information (NCBI) Gene Expression Omnibus (GEO) database (accession numbers GSE27342). The *NKX6.3* gene expression was consistently down-regulated in the gastric cancer cohorts (Figure 1F).

### NKX6.3 inhibits cell proliferation

**Figure 2:**
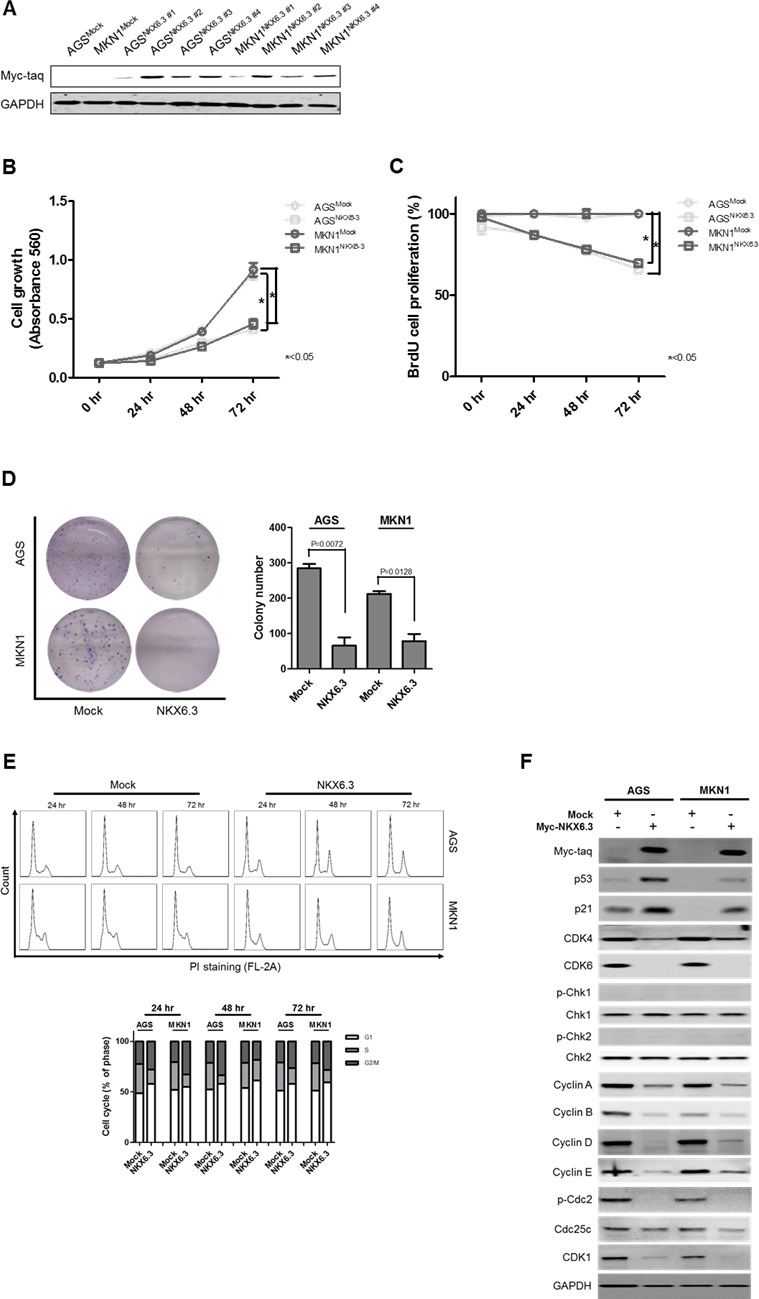
Effects of NKX6.3 on cell viability, cell proliferation, and cell cycle **A.** Stable NKX6.3 transfectants, AGS^NKX6.3^ and MKN1^NKX6.3^, showed marked expression of NKX6.3 by western blot analysis. **B.** and **C.** Cell viability and proliferation were measured by MTT and BrdU incorporation assays in AGS^Mock^, MKN1^Mock^, AGS^NKX6.3^ and MKN1^NKX6.3^ cells. NKX6.3 stable cells demonstrated a time-dependent inhibition of cell viability (B) and proliferation (C). **D.** NKX6.3 stable cells showed significantly reduced colony formation. **E.** NKX6.3 stable cells increased cell population in the G1 and G2/M phases. **F.** NKX6.3 increased p53 and p21 expression, but reduced the expression of positive cell cycle regulators, including CDK4/6, CDK1, Cyclin A, D, E, B, Cdc25c, and p-Cdc2 in gastric cancer cells. However, NKX6.3 did not affect the expression of Chk1 and Chk2.

As shown in Figure 2A, stable NKX6.3 transfectants of AGS^NKX6.3^ and MKN1^NKX6.3^ cells showed marked expression of the NKX6.3 protein relative to the empty mock stable cells, AGS^Mock^ and MKN1^Mock^. Cell viability and proliferation decreased dramatically in AGS^NKX6.3^ and MKN1^NKX6.3^ cells in a time-dependent manner (Figure 2B and 2C). In addition, AGS^NKX6.3^ and MKN1^NKX6.3^ cells significantly reduced the number and size of surviving colonies compared with AGS^Mock^ and MKN1^Mock^ cells (Figure 2D), suggesting that the NKX6.3 expression efficiently arrests cellular proliferation.

### NKX6.3 induces G0/G1 and G2/M arrests

Next, we examined the potential mechanisms underlying the NKX6.3-induced inhibition of cell proliferation. As shown in Figure 2E, NKX6.3 expression had a modest effect on the G1 and G2/M cell cycle progression.

For the G1 arrest, NKX6.3 up-regulated p53 and p21 expression and down-regulated CDK4/6, Cyclin D, and Cyclin A expression (Figure 2F). Additionally, NKX6.3 suppressed the expression of p-Cdc2, Cyclin E, Cyclin B, Cdc25c, and CDK1 proteins at G2/M transition in both stable cells. However, NKX6.3 did not affect Chk1 and Chk2 expression (Figure 2F). These results suggest that NKX6.3 may suppress gastric tumorigenesis by inhibiting cell cycle progression.

### NKX6.3 induces apoptosis

**Figure 3:**
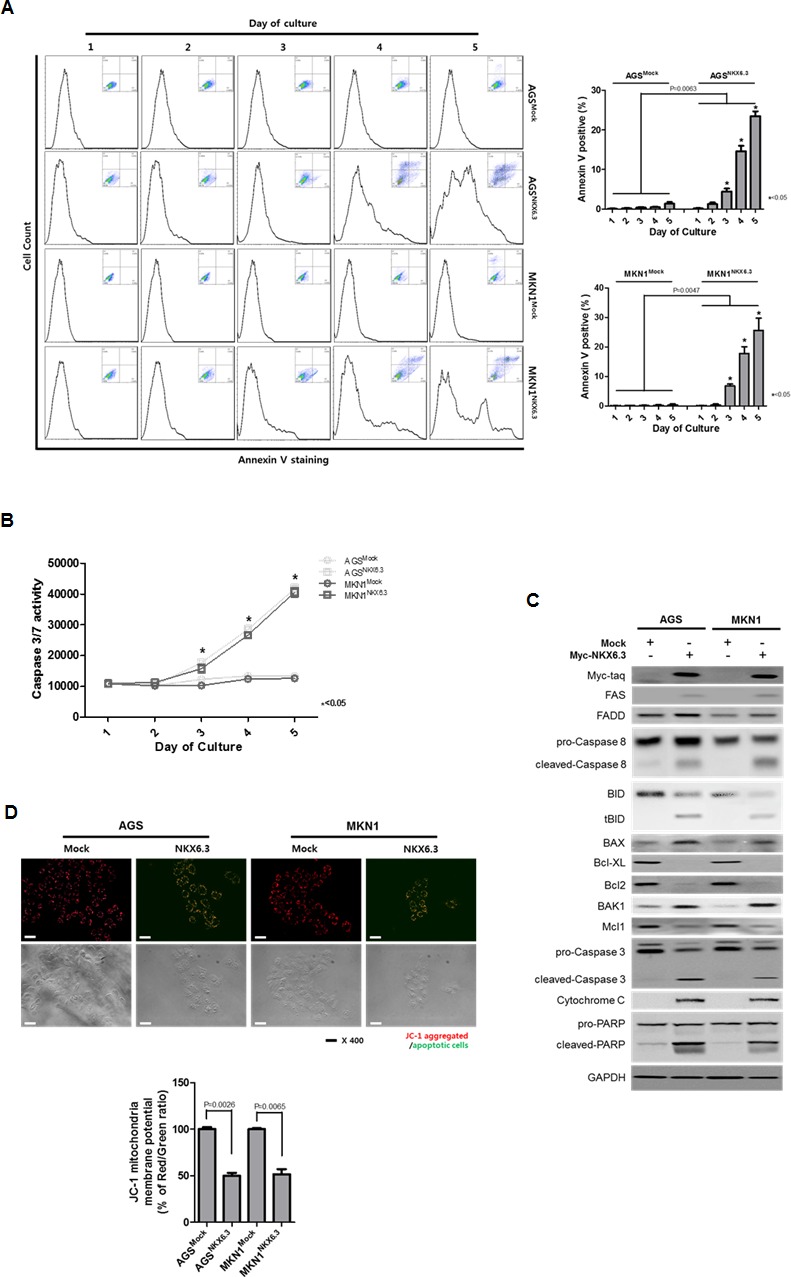
Effects of NKX6.3 on cell death **A.** and **B.** Apoptosis was measured by annexin V-binding and Caspase 3/7 activity assays in AGS^Mock^, MKN1^Mock^, AGS^NKX6.3^ and MKN1^NKX6.3^ cells at 1 to 5 days after seeding. There was a significant increase in annexin V staining cells (A) and Caspase 3/7 activity (B) in AGS^NKX6.3^ and MKN1^NKX6.3^ cells, compared to those in AGS^Mock^, MKN1^Mock^ cells. **C.** NKX6.3 increased the expression of FAS, FADD, truncated BID, BAX, BAK1 and Cytochrome C and also decreased the expression of Bcl-XL, Bcl-2 and Mcl-1. **D.** Both NKX6.3 stable cells demonstrated reduced mitochondrial membrane potentials, as determined by JC-1 staining. JC-1 formed red aggregates in stable mock cells, but remained red and green monomers in stable NKX6.3 cells. We repeated the experiments twice and found that the data were consistent.

NKX6.3 induced apoptosis in a time-dependent manner in AGS^NKX6.3^ and MKN1^NKX6.3^ cells, compared with AGS^Mock^ and MKN1^Mock^ (Figure 3A). Caspase 3/7 activity was significantly enhanced in a time-dependent manner. Additionally, cleaved forms of Caspase 8, Caspase 3, and PARP were found in AGS^NKX6.3^ and MKN1^NKX6.3^ cells (Figure 3B and 3C). Increased expression of FAS, FADD, truncated BID, BAX, BAK1 and cytochrome C as well as decreased expression of Bcl-XL, Bcl-2 and Mcl-1 were detected (Figure 3C). Next, we performed a JC-1 staining assay to determine the effect of NKX6.3 on mitochondrial membrane potential. AGS^Mock^ and MKN1^Mock^ cells stained with JC-1 showed red fluorescence, whereas AGS^NKX6.3^ and MKN1^NKX6.3^ cells exhibited a heterogeneous staining with both red and green fluorescence coexisting in the same cells (Figure 3D).

### NKX6.3 regulates gastric differentiation

**Figure 4:**
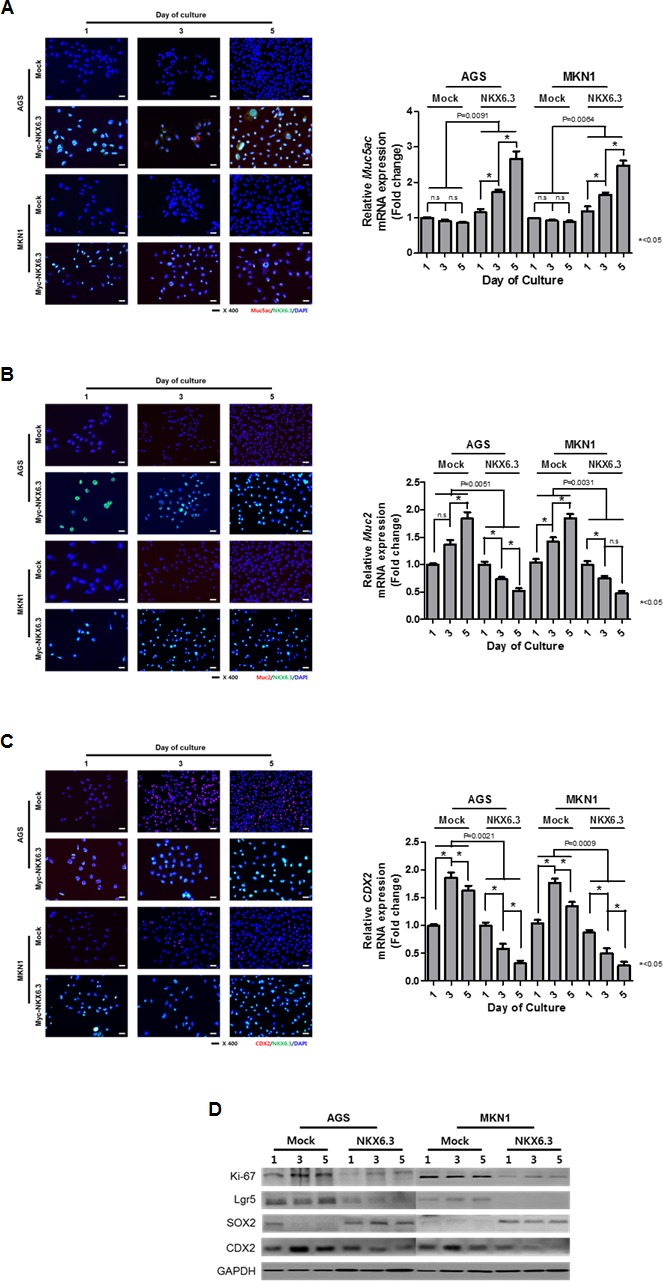
NKX6.3 regulated gastric cell differentiation **A.** Stably *NKX6.3*-transfected cells induced the protein and mRNA expression of gastric foveolar mucin, Muc5ac. **B.** and **C.** NKX6.3 reduced the expression of Muc2 (B) and CDX2 (C) protein and mRNA transcripts. **D.** NKX6.3 down-regulated the expression of Ki-67, Lgr5, and CDX2 proteins, but up-regulated the SOX2 gastric marker in western blot analysis.

Next, we investigated whether NKX6.3 regulates the expression of gastric foveolar mucin Muc5ac, goblet cell mucin Muc2, and CDX2 genes using immunofluorescent analysis and real-time RT-PCR. Expectedly, NKX6.3 increased protein and mRNA expression of Muc5ac (Figure 4A) and reduced expression of Muc2 and CDX2 (Figure 4B and 4C) in a time-dependent manner in both NKX6.3 stable cells, suggesting that NKX6.3 may induce gastric differentiation and inhibit intestinal differentiation.

To further confirm these data, we examined the expression of proteins that induce intestinal differentiation at 1, 3 and 5 days after cell culture. Expectedly, NKX6.3 stable transfectants showed increased expression of the gastric marker SOX2 and reduced expression of the Ki-67, Lgr5, and CDX2 proteins (Figure 4D). These results indicate that NKX6.3 may modulate gastric and intestinal markers.

### Anti-NKX6.3 ChIP-cloning followed by sequencing

**Figure 5:**
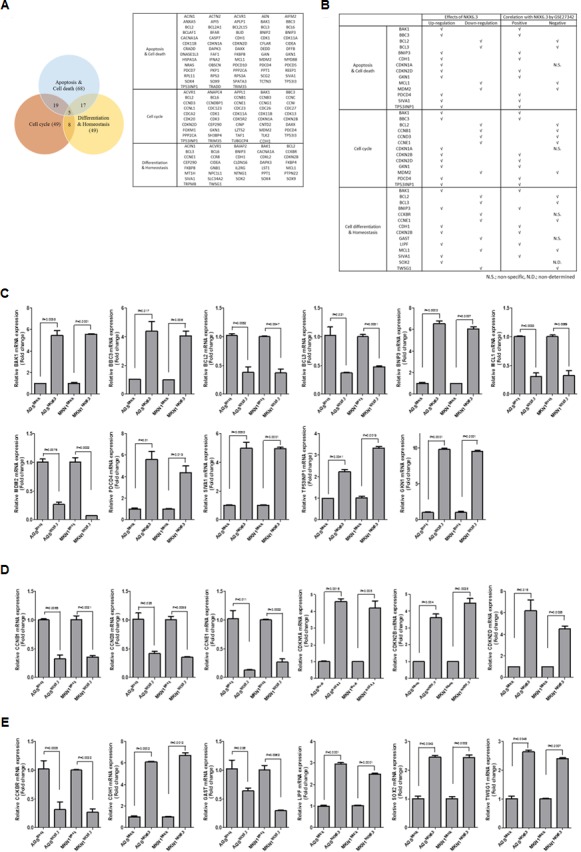
Predicted target genes of NKX6.3 **A.** Venn diagram and list of predicted target genes of NKX6.3 in cell death, cell cycle and differentiation pathways. **B.** Gene expression patterns of NKX6.3, and correlation between predicted target genes and NKX6.3 mRNA expression in the large cohort of gastric cancer patients (GSE27342). **C.**-**E.** NKX6.3-induced changes in mRNA expression of cell death- (C), cell cycle- (D) and differentiation (E)-related genes were confirmed by real-time QPCR.

To investigate the binding profile of NKX6.3, ChIP cloning and sequencing were performed. After sequencing, we considered the genes containing predicted binding motif sequences (TAAT) of *NKX6.3* [9] in the promoter region as candidate target genes of NKX6.3. In addition, since NKX6.3 functions as a master regulator in gastric epithelium, we sorted the genes based on their possible functions (Figure 5A). The genes associated with cell proliferation, death, and differentiation in gastric mucosal epithelium were chosen (Figure 5B). Significant expression changes were verified by real time RT-PCR (Figure 5C–5E).

### NKX6.3 induces GKN1 expression

**Figure 6:**
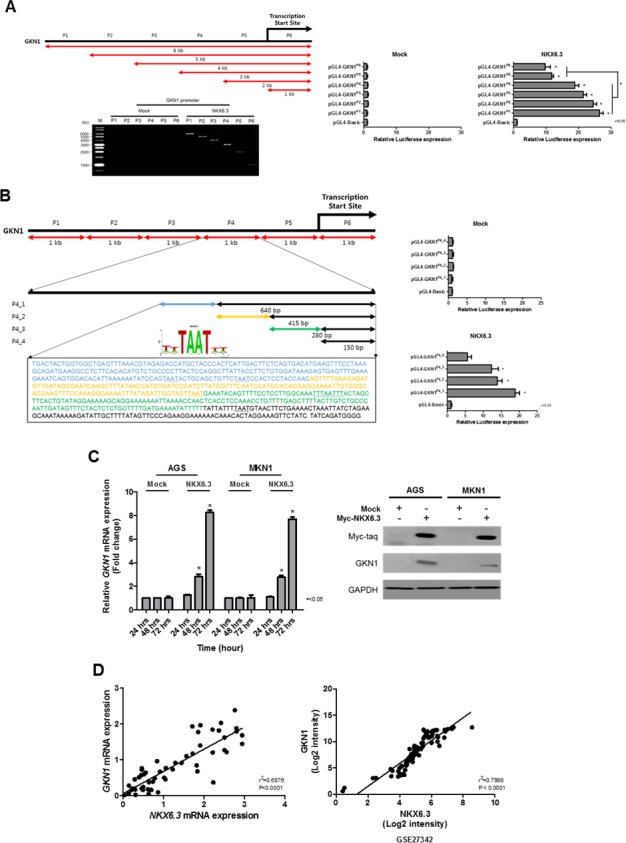
Effects of NKX6.3 on GKN1 expression **A.** Putative promoter activity was characterized between −5 kb and +1 kb relative to the transcription start site (TSS) of GKN1 by ChIP and QPCR. Binding activity of NKX6.3 was detected in the *GKN1* promoter region. Luciferase activity of AGS^NKX6.3^ cells transfected with plasmids with full-length (−5 to +1 kb) or deletions of the *GKN1* promoter containing TSS designated 0 kb, then cultured for 24 h. Luciferase activity showed that NKX6.3 occupancy was 20-fold enriched at the P4 region (positions −2 to +1 kb) compared to the control. Normalized luciferase activity values for each construct (*N* = 3, *<0.05, *t*-test) are represented as mean ± SD. **B.** Five putative NKX6.3 binding motifs were found at the P4 promoter region. Luciferase activity analysis of 5′-deletion constructs at the P4 promoter region showed a significant decrease in promoter activity. Further deletion of the 5′ binding motifs resulted in a progressive loss of activity, indicating that NKX6.3 binding motifs at the P4 construct are required for the GKN1 transcription. **C.** NKX6.3 induced GKN1 mRNA and protein expression in AGS^NKX6.3^ and MKN1^NKX6.3^ cells. **D.** There was positive correlation between NKX6.3 and GKN1 expression in 55 non-cancerous gastric mucosa and large cohort of gastric cancer patients (NCBI GEO database, accession numbers GSE27342).

Interestingly, NKX6.3 dramatically increased the expression of GKN1, a gastric-specific tumor suppressor, at the mRNA level (Figure 5A). To identify a NKX6.3 binding site within the *GKN1* gene promoter upstream sequences, chromatin immunoprecipitation (ChIP) assay followed by PCR was performed in AGS^Mock^, MKN1^Mock^, AGS^NKX6.3^ and MKN1^NKX6.3^ cells. We defined the region upstream of the *GKN1* gene (between −5 kb and +1 kb), overlapping with the transcription start site (TSS) designated 0 kb. We constructed 6 reporter vectors containing 6 kb fragments in positions P1 (−5 to +1 kb), P2 (−4 to +1 kb), P3 (−3 to +1 kb), P4 (−2 to +1 kb), P5 (−1 to +1 kb), and P6 (TSS to +1 kb). Each fragment contained 18, 14, 10, 9, 4, and 3 putative NKX6.3 binding motifs. As shown in Figure 6A, NKX6.3 binding activity was detected from −5 kb to +1 kb relative to the *GKN1* TSS in AGS (Figure 6A) and MKN1 cells (Supplementary Figure 2A). Luciferase assay was performed to determine more precisely the location of the NKX6.3 transcription factor binding within the enriched fragment. NKX6.3 occupancy was 20-fold enriched at the P4 region compared to the control (Figure 6A and Supplementary Figure 2B). Thus, the P4 promoter region was subdivided into 640, 415, 280 and 150-bp fragments containing these binding motifs (Figure 6B), and the appropriate primers were designed (Supplementary Table 1). Significant enrichments of 18, 14, and 12-fold were observed for the fragment containing the first 4 binding motifs, whereas a weak 4-fold enrichment was observed for the fragment containing the 5^th^ binding motif (Figure 6B and Supplementary Figure 2C). In line with the results of ChIP and luciferase assays described above, NKX6.3 dramatically increased the expression of GKN1 at the mRNA and protein levels (Figure 6C). There was a positive correlation between NKX6.3 and GKN1 in non-cancerous gastric mucosal tissues and in the NCBI GEO database (accession numbers GSE27342) (Figure 6D). These findings suggest that NKX6.3 may positively control the expression of GKN1.

**Figure 7:**
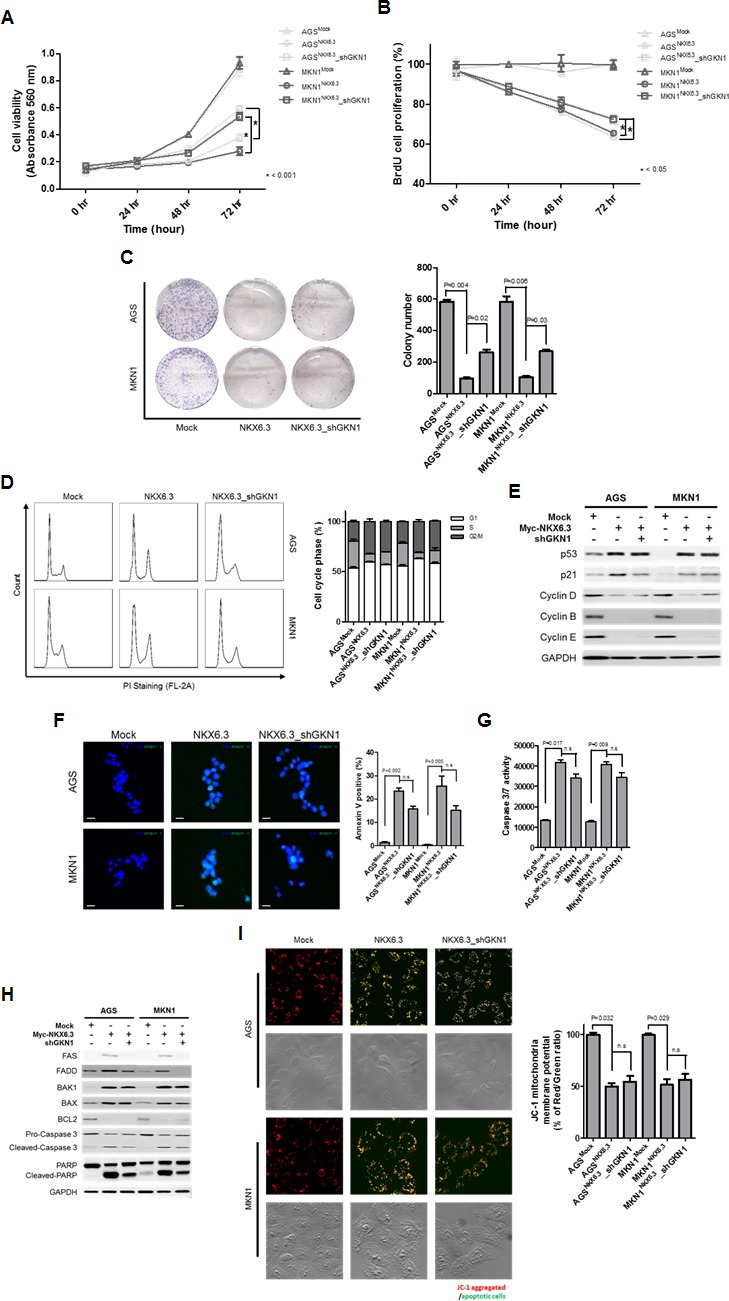
The role of GKN1 in NKX6.3-induced anti-cancer effects **A.**-**C.** GKN1 silencing resulted in a partial inhibition of the NKX6.3-induced anti-cancer effects on cell viability (A), proliferation (B), and colony formation (C). **D.** and **E.** GKN1 silencing partially inhibited the NKX6.3 impacts on cell cycle progression by modulating cell cycle regulatory components. **F.**-**I.** GKN1 silencing partially inhibited the effects of NKX6.3 on cell death by suppressing the death receptor pathway, but it did not affect cell death through the mitochondrial pathway.

To determine whether the tumor suppressor effect of NKX6.3 is dependent on GKN1, we analyzed cell viability and proliferation in AGS^NKX6.3^ and MKN1^NKX6.3^ cells after GKN1 silencing with *shGKN1* (Supplementary Figure 3). GKN1 silencing partially inhibited the effects of NKX6.3 on cell viability, proliferation, colony formation, and cell cycle progression (Figure 7A–7E). However, GKN1 silencing did not affect NKX6.3-induced apoptosis, Caspase 3/7 activity and expression of mitochondrial pathway-related proteins or mitochondrial membrane potentials (Figure 7F–7I). Collectively, these results suggest that NKX6.3 may inhibit cell proliferation and induce apoptosis in a GKN1-dependent or -independent manner.

## DISCUSSION

Loss of tissue-specific differentiation processes is necessary for tumors to reach a primitive and poorly differentiated state [10]. Here, we found loss or reduced expression of NKX6.3 protein in AGS, MKN1, MKN28, and MKN45 gastric cancer cell lines and 33 (94.3%) of 35 gastric cancer tissues (Figure 1A and 1B). To determine whether reduced NKX6.3 expression is caused by somatic or epigenetic changes of the *NKX6.3* gene, we performed mutational and methylation studies of *NKX6.3* gene. There were no somatic mutation and hypermethylation of the gene (Figure 1C), suggesting that genetic and epigenetic alterations of *NKX6.3* may not play an important role in the development of gastric cancer. To further identify the mechanism underlying NKX6.3 inactivation in gastric cancers, DNA copy number and mRNA transcript levels of the *NKX6.3* gene were examined by real-time QPCR and real-time RT-PCR, respectively. Interestingly, markedly decreased DNA copy number and mRNA transcript expression of *NKX6.3* were found in 18 (32.7%) and 34 (61.8%) of 55 gastric cancers, respectively (Figure 1D and 1E). Additionally, frequent allelic loss (52.2%) at the *NKX6.3* locus was detected in gastric cancers (Supplementary Figure 1). These results are consistent with previous reports describing 30% of loss of heterozygosity at chromosome 8p11.21 [8], in which *NKX6.3* resides, as well as reduced *NKX6.3* mRNA expression in gastric cancers detected by microarray analysis [11]. When we compared the DNA copy number with mRNA transcript expression of *NKX6.3*, there was a positive correlation between two variables in non-cancerous gastric mucosa and cancer tissues (*P* < 0.0001) (Figure 1F). Furthermore, NKX6.3 expression was significantly down-regulated in gastric cancer cohorts (Figure 1F). These data suggest that decreased DNA copy number and mRNA transcript of the *NKX6.3* gene may be a driving event behind NKX6.3 inactivation in gastric cancer.

Next, we asked whether NKX6.3 could control cell growth and death. Expectedly, stable expression of NKX6.3 showed a time-dependent inhibition of cell viability, proliferation and colony formation in AGS^NKX6.3^ and MKN1^NKX6.3^ cells (Figure 2B–2D). There was a concurrent increase of G1 and G2/M phases in AGS^NKX6.3^ and MKN1^NKX6.3^ cells (Figure 2E). NKX6.3 selectively induced p53 and p21 expression and elicited concomitant suppression of CDK4, CDK6, Cyclin A, and Cyclin D expression in the G1/S transition. It also triggered suppression of p-Cdc2, Cdc25c, CDK1, Cyclin B, and Cyclin E expression in the G2/M transition, but did not affect the expression of Chk1 and Chk2, key components of the DNA damage signaling network (Figure 2F). These results suggest that NKX6.3 may function as a tumor suppressor by inhibiting cell cycle progression in gastric cancer.

We also found that stable expression of NKX6.3 induces apoptosis and enhances Caspase 3/7 activity in AGS^NKX6.3^ and MKN1^NKX6.3^ cells in a time-dependent manner (Figure 3A and 3B). In addition, expression of apoptosis-related proteins, including FAS, FADD, cleaved-Caspase 8, tBID, BAX, Cytochrome C, cleaved-Caspase 3 and PARP, was detected (Figure 3C). Furthermore, NKX6.3 significantly reduced mitochondrial membrane potential in JC-1 staining assay (Figure 3D). Thus, we concluded that NKX6.3 may stimulate cell death through both death receptor- and mitochondrial-pathways. Together with the results presented in Figure 3, our data indicate that NKX6.3 may function as a tumor suppressor for gastric cancer by inducing apoptosis as well as cell cycle arrest.

In mice, NKX6.3 expression was detected in the glandular stomach, which segregates the lower/base region of the gastric unit, and its expression was activated in the course of differentiation in the antral part of the mouse stomach [6, 7]. In the human stomach, NKX6.3 expression was observed in the corpus and antrum, which are covered by mucous-secreting columnar epithelium (Figure 1A). Notably, NKX6.3 induced the expression of gastric foveolar mucin Muc5ac at the protein and mRNA level (Figure 4A), while it reduced the expression of the goblet cell mucin Muc2 and CDX2, an intestinal-specific homeobox transcription factor (Figure 4B and 4C). It is widely known that altered expression of Muc5ac with the aberrant expression of Muc2 and CDX2 was detected in intestinal metaplasia of the stomach, which is considered to be a preneoplastic stage of gastric carcinogenesis [12–15]. Interestingly, NKX6.3 down-regulated the expression of Ki-67 (proliferative marker), Lgr5 and CDX2 (intestinal markers), and up-regulated expression of the gastric marker SOX2 (Figure 4D). These results are consistent with the observation that cells expressing the mutant *NKX6.3* gene may represent immature cells [7] and suggest that NXK6.3 may play an important role in gastric homeostasis and tumorigenesis by inducing gastric differentiation and preventing intestinal differentiation in gastric mucosal epithelial cells. Therefore, it is likely that NKX6.3 depletion may render gastric mucosal epithelial cells subject to subsequent genetic or epigenetic alterations in tumor suppressor genes or oncogenes.

Targets of the NKX6.3 transcription factor in the gastric mucosal epithelium are poorly characterized. In ChIP-cloning and sequencing analyses, we identified a repertoire of candidate NKX6.3 target genes involved in regulation of the cell cycle, death and differentiation (Figure 5). We confirmed by real-time RT-PCR that NKX6.3 coordinates the expression of these genes, including GKN1, in AGS^NKX6.3^ and MKN1^NKX6.3^ cells (Figure 5). Previously, we demonstrated that GKN1 inactivation is frequently detected in gastric cancers and that GKN1 inhibits cell proliferation while inducing senescence and apoptosis [16–19]. Thus, we investigated whether NKX6.3 functions as a transcriptional factor for GKN1. Reporter gene assay showed that NKX6.3 occupancy was 20-fold enriched at the P4 region containing 9 putative NKX6.3 binding motifs (TAAT) (Figure 6A and Supplementary Figure 2A and 2B). Further deletion of the 5′ sequence resulted in progressive loss of promoter activity. The constructs containing the first 4 binding motifs resulted in a significant increase in promoter activity up to 18 fold (Figure 6B and Supplementary Figure 2C), indicating that NKX6.3 binding motifs in this region are required for transcriptional up-regulation of GKN1. Expectedly, GKN1 expression was affected at the mRNA and protein levels in only AGS^NKX6.3^ and MKN1^NKX6.3^ cells (Figure 6C). We therefore concluded that NKX6.3 functions as a master regulator of gastric mucosal epithelial cells by regulating the expression of cell fate-related proteins and that GKN1 is one of the transcription targets of NKX6.3.

Next, we asked whether the NKX6.3 activity is dependent on the GKN1. Interestingly, treatment with *shGKN1* showed partial ablation of the NKX6.3-induced growth-inhibitory activity (Figure 7A–7E). However, GKN1 silencing did not affect NKX6.3 induced apoptosis, Caspase 3/7 activity and mitochondrial membrane potential (Figure 7F–7I), suggesting that NKX6.3 may activate not only the death receptor, but also the mitochondrial pathway. Overall, these data indicate that NKX6.3 employs GKN1-dependent and -independent mechanisms for the regulation of cell cycle and death.

In conclusion, complete loss or significantly reduced expression of NKX6.3 protein was frequently observed in gastric cancers. Decreased DNA copy number and mRNA transcript of the *NKX6.3* gene was a driving force behind NKX6.3 inactivation in gastric cancers. In functional analysis, NKX6.3 markedly arrested cell proliferation by inhibiting cell cycle progression and induced apoptosis through both death receptor- and mitochondrial-pathways. In addition, NKX6.3 increased expression of the gastric differentiation markers, including SOX2 and Muc5ac, and decreased expression of the intestinal differentiation markers, CDX2 and Muc2. Furthermore, NKX6.3 regulated a repertoire of target genes associated with cell fate resulting in gastric differentiation, inhibition of cell proliferation and induction of apoptosis in GKN1-dependent and independent manners. Thus, we conclude that the *NKX6.3* gene may play an important role in the development of gastric cancer, acting as a tumor suppressor.

## MATERIALS AND METHODS

### Human gastric samples

A total of 55 frozen gastric cancers were obtained from the Chonnam National University Hwasun Hospital, which is supported by the Ministry of Health, Welfare and Family Affairs. Informed consent was provided according to the Declaration of Helsinki. Written informed consent was obtained from all subjects. The study was approved by the Institutional Review Board of The Catholic University of Korea, College of Medicine (MC15SISI0015). There was no evidence of familial cancer in any of the patients.

### Mutational and methylation analyses of the *NKX6.3* gene in gastric cancers

Genomic DNAs from each tumor and corresponding non-cancerous gastric mucosal cells were amplified with 6 sets of primers covering the entire coding region of the *NKX6.3* gene. The specific primers for detection of *NKX6.3* mutation were designed according to the genomic sequence of Genbank accession No. NC_000008.11. The primer sequences are described in Supplementary Table 2. Each polymerase chain reaction (PCR) procedure was performed, as described in the literature with minor modifications [16].

Methylation status of the promoter region of the *NKX6.3* gene was determined using sodium bisulfite treatment of DNA followed by MSP, as described in the literature with minor modifications [16].

### DNA copy number changes and mRNA expression of *NKX6.3* in gastric cancers

After quantification of genomic DNA and mRNA extracted from gastric cancers and corresponding non-cancerous gastric mucosae, real-time SYBR Green QPCR was performed on a Stratagene Mx 3000P QPCR system. Specific primers for detection of the *NKX6.3* DNA copy number were designed according to the genomic sequence of Genbank accession No. NC_000008.11. All samples were subjected to PCR amplification with oligonucleotide primers specific for the constitutively expressed gene, glyceraldehyde-3-phosphate dehydrogenase (*GAPDH*), and normalized. Primers for SYBR Green analysis were designed based on the gene-specific non-homologous DNA sequences. The primer sequences are described in Supplementary Table 2.

cDNA was synthesized using the reverse transcription kit from Roche Molecular System (Roche, Mannheim, Germany), according to the manufacturer’s protocol. For QPCR, 50 ng cDNA was amplified using Fullvelocity SYBR Green QPCR Master Mix (Stratagene, La Jolla, CA, USA) and 20 pmol/μl of each primer (forward and reverse) using Stratagene Mx 3000P QPCR system, according techniques previously published [16]. Due to low Ct value, real-time RT PCR was carried out for 45 cycles. To ensure the fidelity of mRNA extraction and reverse transcription, all samples were subjected to PCR amplification with oligonucleotide primers specific for the constitutively expressed gene, glyceraldehyde-3-phosphate dehydrogenase (*GAPDH*), and normalized. The standard curve method was used for quantification of the relative amounts of gene expression products. This method provides unit-less normalized expression values that can be used for direct comparison of the relative amounts of target DNA and mRNA in different samples. All samples were tested in duplicate, and the average values were used for quantification. Reduced DNA copy number and mRNA expression were defined as a mean test (cancer)/reference (mucosa) ratio.

### Loss of heterozygosity (LOH) analysis for the *NKX6.3* gene locus

We analyzed allelic loss with two microsatellite markers, D8S464 and D8S2329. DNAs extracted from tumor and corresponding non-cancerous gastric mucosa were amplified using a thermal cycler (MJ Research Institute, Watertown, MA, USA) with the two microsatellite markers, as described previously [20]. The PCR products were loaded onto a SSCP gel (FMC Mutation Detection Enhancement system; Intermountain Scientific, Kaysville, UT, USA) containing 10% glycerol. Complete absence or at least 50% reduced intensity of one allele in the tumor DNA of the informative cases was considered as LOH. To confirm theses results, sequencing of the PCR products was carried out using an ABI-377 automated fluorescent DNA sequencer (Applied Biosystems, Warrington, UK), according to the manufacturer’s recommendations.

### Cell culture and transfection of NKX6.3

AGS and MKN1 gastric cancer cells lines were cultured at 37°C in 5% CO_2_ in RPMI-1640 medium with 10% heat-inactivated fetal bovine serum. Complete *NKX6.3*-cDNA was cloned into the expression vector pCMV6-Myc-DDK (Origene). AGS and MKN1 cells were transiently transfected with expression plasmids (5 μg total DNA) in 60 mm-diameter dishes using Lipofectamine Plus transfection reagent (Invitrogen), according to the manufacturer’s recommendations.

We also generated stable NKX6.3 transfectants of AGS and MKN1 cells, AGS^NKX6.3^ and MKN1^NKX6.3^, stably expressing human NKX6.3, as well as mock transfectants, AGS^Mock^ and MKN1^Mock^ cells, as described previously [18]. Stable expression of NKX6.3 was confirmed in AGS^NKX6.3^ and MKN1^NKX6.3^ cells by western blot analysis.

### Measurement of cell viability, proliferation, and colony formation

Cell viability, proliferation, and colony formation were analyzed using MTT, BrdU and clonogenic assays, as described previously [16–18].

### Measurement of apoptosis and flow-cytometric analysis of the cell cycle

For apoptosis assessment, annexin V-binding assay was performed at 1 to 5 days in AGS^Mock^, MKN1^Mock^, AGS^NKX6.3^ and MKN1^NKX6.3^ cells, as described previously [21]. To confirm whether NKX6.3 induces apoptosis and the effects of GKN1 on NKX6.3-induced apoptosis caused by caspase activation, we examined Caspase-3 and -7 activity using an Apo-One Homogeneous Caspase 3/7 assay kit (Promega) as described previously [21].

For measurement of mitochondrial membrane potential, cells were stained with the cationic dye JC-1 (MitoPT, Immunohistochemistry Technologies), which exhibits potential-dependent accumulation in mitochondria.

For cell cycle analysis, the cells from each experimental group were collected and stained with PI for 45 min in the dark before analysis. The percentages of cells in different phases of the cell cycle were determined using a FACSCalibur Flow Cytometer with CellQuest 3.0 software (BD Biosciences).

### Reverse transcription and real-time PCR

Total RNA was extracted following the TRIzol Reagent (Invitrogen) protocol. Two micrograms of total RNA was used in reverse transcription following the Superscript III (Invitrogen) protocol. Quantitative PCR was performed on an IQ5 optical system (Bio-rad) using SYBR Green Q-PCR Master Mix (Bio-rad), according to the manufacturer’s instructions. Primer sequences of the genes are described in Supplementary Tables 3-4. Gene expression data were normalized to *GAPDH*.

### Immunoblot and Immunofluorescence (IF)

The following antibodies were used: NKX6.3, GKN1, Lgr5, SOX2 (Abcam), FAS, FADD, Caspase 8, BID, BAX, Bcl-XL, Bcl2, BAK1, Mcl-1, Caspase 3, PARP, Cytochrome C, p53, p21, CDK4/6, Cyclin D1, Chk1, Chk2, p-Cdc2, Cyclin A, Cyclin B, Cyclin E, Cdc25c, CDK1 (Cell signaling), Muc5ac, Muc2 (Leica), CDX2 (Biogenetex), Ki-67 (Santacruz).

### Chromatin immunoprecipitation (ChIP)

For assessing the NKX6.3 binding activity in the promoter region of *GKN1*, ChIP assays were performed using the Thermo Scientific Pierce Agarose ChIP kit (Thermo Scientific Pierce), as described previously [18]. DNA amplification was performed by PCR using primers for the *GKN1* promoter described in Supplementary Table 1. Amplification products were separated on a 2% agarose gel.

### Cloning of the ChIP fragments

The immunoprecipitated DNA was cloned as described previously [22]. Briefly, the DNA isolated from ChIP was heated at 68°C for 5 min and then cooled to 37°C. One to two units of T4 DNA polymerase were added to the DNA and reaction mix containing repair buffer (18 mM ammonium sulfate, 66 mM Tris [pH 8.0], 6.6 mM MgCl_2_, 50 mM β-mercaptoethanol, and 0.5 mM of each nucleotide) and subsequently incubated at 37°C for 15 min. The reaction was terminated by 1 μl of 0.5 M EDTA for a 50 μl reaction mix. The processed DNA was cloned into pUC118 Hinc II/BAP vector (Takara). Each ligation was transformed into DH-5α competent cells (Clontech). The entire transformation was plated onto ampicillin treated Luria broth agar plates. Randomly picked 120 colonies with inserts were identified by PCR using M13 primers spanning the cloning site in the vector. Inserts > 200 bp were selected for sequencing using a capillary automatic sequencer (3730 DNA Analyzer, Applied Biosystem). The BLAST search of the human genome database at NCBI was performed to locate sequences. The possible genomic binding sequences were identified by pattern matching. Specific sequences were also analyzed using BLAST adjusted to short sequences (Program = blastn, Word size = 7, Expect Value = 100, Filter = disabled). The sequence logo was generated by WebLogo (http://weblogo.berkeley.edu/logo.cgi).

### NKX6.3 binding site in a luciferase reporter assay

To assess the quality of the ChIP, NKX6.3 occupancy was detected by qPCR at the *GKN1* promoters. Primers were designed and verified to produce one amplicon on genomic DNA. Primer sequences were described in Supplementary Table 1.

The pGL4.10[luc2] vector (Promega), containing the gene for luciferase, was used in this study. To create a pGL4.10-GKN1 promoter construct containing the human *GKN1* promoter upstream to the luciferase gene, the *GKN1* promoter was amplified by PCR from human genomic DNA. The GKN1 promoter primers were described in Supplementary Table 1. PCR products were digested with *Kpn*I and *Xho*I enzymes and then ligated into a pGL4.10[luc2] vector that was digested with the same enzymes. Luciferase reporter assay was performed using a Dual-Luciferase Reporter Assay System (Promega) following the manufacturer’s protocol.

### Statistical analysis

Student’s *t*-test was used to analyze the effect of NKX6.3 on cell viability. Data are expressed as means ± S.D. from at least three independent experiments. The linear regression, correlation, and Chi-square tests were used to examine the association of DNA copy number change with mRNA and protein expression of the *NKX6.3* gene. A *P*-value less than 0.05 was considered to be the limit of statistical significance.

## SUPPLEMENTARY MATERIAL FIGURES AND TABLES


