# Supplementary material for: NKX6.3 controls gastric differentiation and tumorigenesis
Source: Oncotarget. 2015 Jul 22;6(29):28425–39. doi: 10.18632/oncotarget.4952 (PMC4695069; doi:10.18632/oncotarget.4952)
Supplement: Supplementary file 1 [file oncotarget-06-28425-s001.pdf]

# NKX6.3 controls gastric differentiation and tumorigenesis

## Supplementary Material

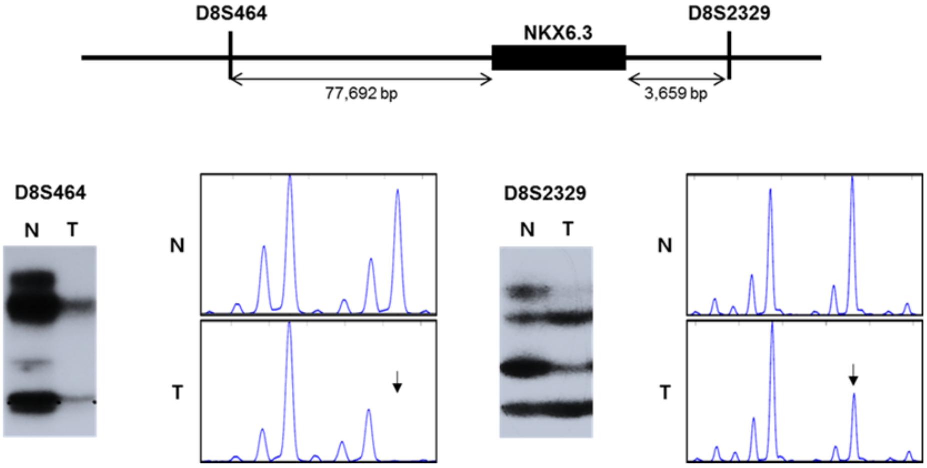


**Supplementary Figure 1.** Allelic loss of NKX6.3 gene in gastric cancer.

Allelic loss of NKX6.3 gene in gastric cancer with microsatellite markers D8S464 and D8S2329, which are located -77.692 kb and +3.659 kb from the *NKX6.3* locus, respectively. Representative autoradiograph and fluorescent microsatellite analysis showed loss of heterozygosity (LOH) at the *NKX6.3* locus.

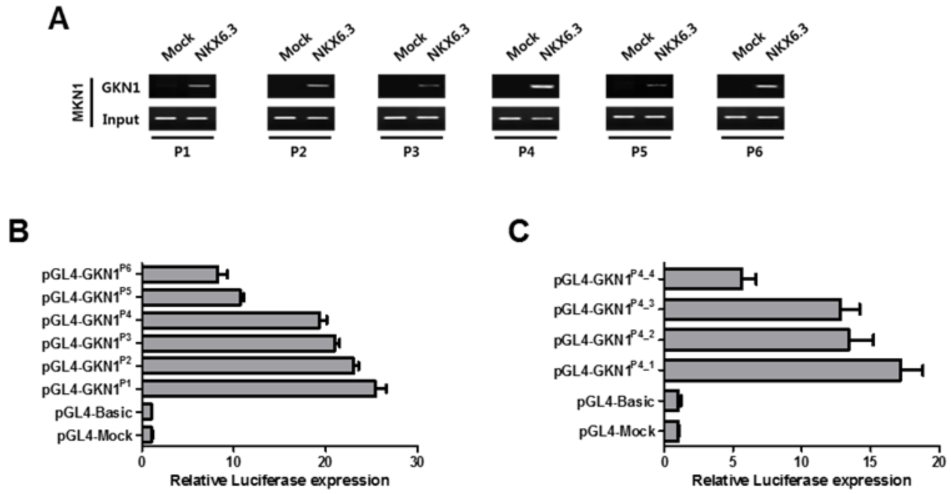


**Supplementary Figure 2. Binding activity of NKX6.3 to the GKN1 promoter.**

**A.** Putative promoter activity was characterized between -5 kb and +1 kb relative to transcription start site (TSS) of GKN1 by ChIP and QPCR.

**B.** Reporter gene luciferase assays showed that NKX6.3 occupancy was 18-fold enriched at the P4 region compared to control. Normalized luciferase activity values for each construct (N=3) are represented as mean ± SD.

**C.** Activity analysis of 5’-deletion constructs at the P4 promoter region demonstrated a significant decrease in promoter activity. Further deletion of the 5’ binding motifs resulted in a progressive loss of activity, indicating that NKX6.3 binding motifs at P4 construct are required for the GKN1 transcription in MKN1 cells.

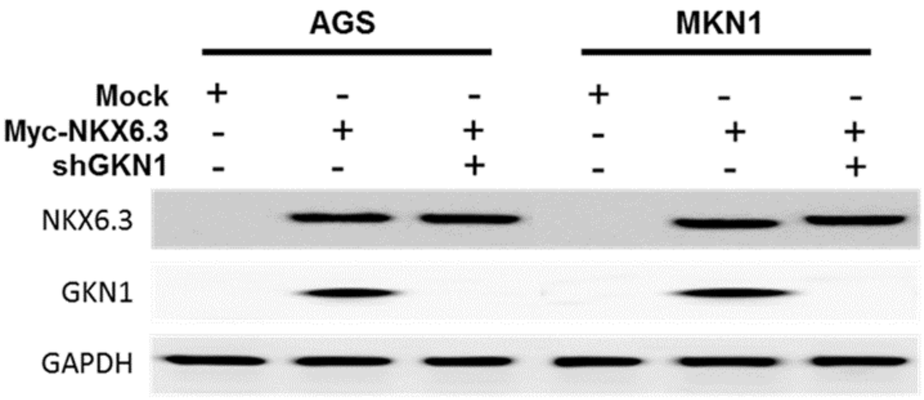


**Supplementary Figure 3. GKN1 silencing by shRNA in NKX6.3 stable cells.**

NKX6.3 induced the GKN1 expression, whereas GKN1 silencing with *shGKN1* completely ablated NKX6.3-induced GKN1 expression.

**Supplementary Table 1.** The primer sequences used for studies of the *NKX6.3* binding activity on *GKN1* promoter region

| Primer name | Sequences |
| --- | --- |
| ChIP-PCR | |
| P1 | F: 5'- GTACCTGAGCATTGTTTGTTTTACATCTT -3'<br>R: 5'- CGAGGCCGTAACGTTTTCCAGTGTGTAG -3' |
| P2 | F: 5'- TACCTCATACACATGATGCAATTACTCA -3'<br>R: 5'- CGAGGCCGTAACGTTTTCCAGTGTGTAG -3' |
| P3 | F: 5'- TACCATCTGATTGACATCTTTAAATGAG -3'<br>R: 5'- CGAGGCCGTAACGTTTTCCAGTGTGTAG -3' |
| P4 | F: 5'- TACCGGAAAACGATCAGGTTCTGTAGTC -3'<br>R: 5'- CGAGGCCGTAACGTTTTCCAGTGTGTAG -3' |
| P5 | F: 5'- TACCAGGAAAGTTCTATCTATCAGATGG -3'<br>R: 5'- CGAGGCCGTAACGTTTTCCAGTGTGTAG -3' |
| P6 | F: 5'- TACCTGACCCCACCTGATAACACCTAGT -3'<br>R: 5'- CGAGGCCGTAACGTTTTCCAGTGTGTAG -3' |
| Luciferase assay | |
| P1 | F: 5'- CTTCGGGGTACCTGAGCATTGTTTGTTTTACATCTTTAAAT -3'<br>R: 5'- GTTGCCCTCGAGGCCGTAACGTTTTCCAGTGTGTAG -3' |
| P2 | F: 5'- CTTCGGGGTACCTCATACACATGATGCAATTACTCAGGA -3'<br>R: 5'- GTTGCCCTCGAGGCCGTAACGTTTTCCAGTGTGTAG -3' |
| P3 | F: 5'- CTTCGGGGTACCATCTGATTGACATCTTTAAATGAGCAG -3'<br>R: 5'- GTTGCCCTCGAGGCCGTAACGTTTTCCAGTGTGTAG -3' |
| P4 | F: 5'- CTTCGGGGTACCGGAAAACGATCAGGTTCTGTAGTCA -3'<br>R: 5'- GTTGCCCTCGAGGCCGTAACGTTTTCCAGTGTGTAG -3' |
| P5 | F: 5'- CTTCGGGGTACCAGGAAAGTTCTATCTATCAGATGGGG -3' |

| | |
|---|---|
| | R: 5’- GTTGCCCTCGAGGCCGTAACGTTTTCCAGTGTGTAG -3’ |
| P6 | F: 5’- CTTCGGGGTACCTGACCCCACCTGATAACACCTAGTT -3’<br>R: 5’- GTTGCCCTCGAGGCCGTAACGTTTTCCAGTGTGTAG -3’ |
| P4_1 | F: 5’- CTTCGGGGTACCTGACTACTGGTGGCTGAGTTTAAAC -3’<br>R: 5’- GTTGCCCTCGAGCCCCATCTGATAGATAGAACTTTCCTA -3’ |
| P4_2 | F: 5’- CTTCGGGGTACCCCAGTTTTGAAGAGATGTTGATAGCGAAT -3’<br>R: 5’- GTTGCCCTCGAGCCCCATCTGATAGATAGAACTTTCCTA -3’ |
| P4_3 | F: 5’- CTTCGGGGTACCCGAAATACAGTTTTCCTCCTTGGCAAA -3’<br>R: 5’- GTTGCCCTCGAGCCCCATCTGATAGATAGAACTTTCCTA -3’ |
| P4_4 | F: 5’- CTTCGGGGTACCCCTACTCTCTGCTTTTGATGAAAATATTTTT -3’<br>R: 5’- GTTGCCCTCGAGCCCCATCTGATAGATAGAACTTTCCTA -3’ |

**Supplementary Table 2.** The primer sequences used for studies of the *NKX6.3* gene

| Primer name | Sequences |
|---|---|
| Mutation analysis | |
| E1-1 | F: 5’- CGGTGTGCCAGTACTCTGTG -3’<br>R: 5’- CCTGGGGGCTGTAGTAGACC -3’ |
| E1-2 | F: 5’- CTCGCAGGGGGTCTACTACA -3’<br>R: 5’- GGACCCGTACTCACTGTTGC -3’ |
| E2 | F: 5’- AACCCCACACTTCCTTCCTC -3’<br>R: 5’- TCTCCCGCACAGACTTACCT -3’ |
| E3-1 | F: 5’- TGTCCTTCCCCAACAGAAAC -3’<br>R: 5’- CTCTTCTTCCGCCACTTGGT -3’ |
| E3-2 | F: 5’- TCTTTCTCATCGCAGGTGTG -3’<br>R: 5’- GATCTTCTCGTCGTCCGAGT -3’ |
| E3-3 | F: 5’- CGAGGACGACGAGTACAACA -3’<br>R: 5’- GAAGGGGAGGGGAAGGTA -3’ |
| Methylation | |
| NKX6.3 M | F: 5’- ATATTTATTGGGTATGATCGAGTCG -3’<br>R: 5’- GTTATATAACCACCTCTCTCCAACG -3’ |
| NKX6.3 U | F: 5’- TATTTATTGGGTATGATTGAGTTGT -3’<br>R: 5’- ATTATATAACCACCTCTCTCCACCAC -3’ |
| DNA copy number | |
| NKX6.3 | F: 5’- AACCCCACACTTCCTTCCTC -3’<br>R: 5’- TCTCCCGCACAGACTTACCT -3’ |
| GAPDH | F: 5’-ACCCAGAAGACTGTGGATGG-3’<br>R: 5’-TTCTAGACGGCAGGTCAGGT-3’ |
| Real time-RT-PCR | |
| NKX6.3 | F: 5’- TCTTTCTGCTTCTGGGGTGT -3’<br>R: 5’- GTCCAGCGGCTTGTTGTACT -3’ |

| | |
|---|---|
| GAPDH | F: 5’-AAATCAAGTGGGGCGATGCTG-3’<br>R: 5’-GCAGAGATGATGACCCTTTTG-3’ |

**Supplementary Table 3.** The primer sequences used for studies of differentiation related gene expresion

| Primer name | Sequences |
|---|---|
| Muc5ac | F: 5’- GCTCAGCTGTTCTCTGGATGAG -3’ |
| | R: 5’- TTACTGGAAAGGCCCAAGCA -3’ |
| Muc2 | F: 5’- CTTCGACGGACTCTACTACAGC -3’ |
| | R: 5’- CTTTGGTGTTGTTGCCAAAC -3’ |
| CDX2 | F: 5’- GCGGAACCTGTGCGAGTG -3’ |
| | R: 5’- GACTGTAGTGAAACTCCTTCTCCAGC -3’ |

**Supplementary Table 4.** The primer sequences used for predicted NKX6.3 target gene expression

| Primer name | Sequences |
|---|---|
| BAK1 | F: 5’- AGAGCTGTCTGAACTCACGT -3’ |
| | R: 5’- TTACACTGTGCCAGAGCCAT -3’ |
| BBC3 | F: 5’- GACCTCAACGCACAGTA -3’ |
| | R: 5’- CTAATTGGGCTCCATCT -3’ |
| BCL2 | F: 5’- GATTGTGGCCTTCTTTGAG -3’ |
| | R: 5’- CAAACTGAGCAGAGTCTTC -3’ |
| BCL3 | F: 5’- GAAAACAACAGCCTTAGCATGGT -3’ |
| | R: 5’- CTGCGGAGTACATTTGCG -3’ |
| BNIP3 | F: 5’- TGGACGGAGTAGCTCCAAGAGC -3’ |
| | R: 5’- AGAAGCCCTGTTGGTATCTTGTG -3’ |
| GKN1 | F: 5’- CAAAGTCGATGACCTGAGCA -3’ |
| | R: 5’- CTTGCCTCTTGCATCTCCTC -3’ |
| MCL1 | F: 5’- AAAGCCTGTCTGCCAAAT -3’ |
| | R: 5’- CCTATAAACCCACCACTC -3’ |
| MDM2 | F: 5’- CAGCAGGAATCATCGGACTCA -3’ |
| | R: 5’- CCTTATTACACACAGAGCCAGGC -3’ |
| PDCD4 | F: 5’- ATGGATATAGAAAATGAGCAGAC -3’ |
| | R: 5’- CCAGATCTGGACCGCCTATC -3’ |
| SIVA1 | F: 5’- TTCAGAACCACACGGCTAC -3’ |
| | R: 5’- TTCCTCTCTTTTTCCTCCC -3’ |
| TP53INP1 | F: 5’- CTTCCTCCAACCAAGAACCA -3’ |
| | R: 5’- GATGCCGGTAAACAGGAAAA -3’ |
| CCNB1 | F: 5’- GGCCAAAATGCCTATGAAGA -3’ |
| | R: 5’- AGATGTTTCCATTGGGCTTG -3’ |

| | |
|---|---|
| CCND3 | F: 5’- CTGGCCATGAACTACCTGGA -3’ |
| | R: 5’- CCAGGAAATCATGTGCAATC -3’ |
| CCNE1 | F: 5’- CCACACCTGACAAAGAAGATGATGAC -3’ |
| | R: 5’- GAGCCTCTGGATGGTGCAATAAT -3’ |
| CDKN1A | F: 5’- GGCAGACCAGCATGACAGATT -3’ |
| | R: 5’- GCGGATTAGGGCTTCCTCT -3’ |
| CDKN2B | F: 5’- CACCGTTGGCCGTAAACTTAAC -3’ |
| | R: 5’- TAATGAAGCTGAGCCCAGGTCT -3’ |
| CDKN2D | F: 5’- TCACACTGCTGTGGTCAGCTT -3’ |
| | R: 5’- CGTCCCTGCGATGGAGAT -3’ |
| CCKBR | F: 5’- TCACACTGCTGTGGTCAGCTT -3’ |
| | R: 5’- CGTCCCTGCGATGGAGAT -3’ |
| CDH1 | F: 5’- GAACAGCACGTACACAGCCCT -3’ |
| | R: 5’- GCAGAACTGTCCCTGTCCCAG -3’ |
| GAST | F: 5’- TCACACTGCTGTGGTCAGCTT -3’ |
| | R: 5’- CGTCCCTGCGATGGAGAT -3’ |
| LIPF | F: 5’- CTCTAGCTCCTGTTGCCACT -3’ |
| | R: 5’- TACACATCCAAGCGACTCGT -3’ |
| SOX2 | F: 5’- TTGCTGCCTCTTTAAGACTAGGA -3’ |
| | R: 5’- CTGGGGCTCAAACTTCTCTC -3’ |
| TWSG1 | F: 5’- TGAGCAAATGCCTCATTCAG -3’ |
| | R: 5’- GGTTGCACATACCGACACAG -3’ |
